# Evaluation of Salivary Alkaline Phosphatase Levels in Passive Smokers of Different Age Groups

**DOI:** 10.7759/cureus.41336

**Published:** 2023-07-03

**Authors:** Sayem A Mulla, Aarti S Bedia, Haritha K Nimmagadda, Sumit Bedia, Amit H Patil

**Affiliations:** 1 Oral Medicine and Radiology, Bharati Vidyapeeth (Deemed to be University) Dental College and Hospital, Navi Mumbai, IND; 2 Anatomy, Bharati Vidyapeeth (Deemed to be University) Dental College and Hospital, Navi Mumbai, IND; 3 Prosthodontics, Bharati Vidyapeeth (Deemed to be University) Dental College and Hospital, Navi Mumbai, IND; 4 Conservative Dentistry and Endodontics, Bharati Vidyapeeth (Deemed to be University) Dental College and Hospital, Navi Mumbai, IND

**Keywords:** salivaomics, salivary alkaline phosphatase, tobacco smoke, secondhand smoke, passive smoking

## Abstract

Background

The smoke inhaled by a nonsmoker from the smoldering end of a cigarette is referred to as passive smoke. The nicotine present in smoke is known to cause tissue damage and alter the enzymatic composition of the body. Alkaline phosphatase (ALP) is a group of intracellular hydrolytic enzymes known to partake in cellular metabolism. ALP levels are affected by smoking as well as passive smoking (PS) with a change in the pH of the oral cavity. The association of salivary alkaline phosphatase (S-ALP) levels in different age groups, gender, and times of exposure is not thoroughly explored yet, which was the primary aim of this study.

Material and methods

A total of 64 samples were collected from passive smokers and non-smokers. Unstimulated saliva (2-2.5 mL) was collected from each subject after obtaining their consent, followed by centrifuging and mixing with ALP reagent in a semi-autoanalyzer to obtain the S-ALP levels.

Results

Higher S-ALP levels were seen in passive smokers (34.70 IU/L) compared to healthy individuals (12 IU/L), which came to be statistically significant (p<0.01). S-ALP levels, when compared to different age groups and gender, were statistically insignificant (p>0.05). However, higher levels were seen in association with time of exposure in passive smokers where the data was statistically significant (p<0.01), suggesting tissue damage possibly due to oxidative stresses and tissue inflammation on continuous exposure for a minimum of 30-60 minutes daily as per our study.

Conclusion

Significantly high levels of S-ALP were found in passive smokers in comparison to non-smokers. This suggests that passive smoking has negative effects on the body tissues. Age, gender, and time of exposure of a non-smoker to tobacco smoke can lead to alterations in S-ALP levels. High levels of S-ALP were seen in individuals with prolonged exposure to tobacco smoke on a daily basis. Salivaomics can thus be used as a non-invasive, economical, and accurate alternative in tissue damage diagnosis.

## Introduction

Passive smoke, also known as secondhand smoke or involuntary smoke, is considered to be an indoor pollutant. It is notorious for being a lethal cocktail of over 4,000 chemicals, 250 of which are said to be harmful with 25 of them being linked to cancers. It is the smoke inhaled by a non-smoker from the smoldering end of a cigarette. It is composed of aged exhaled mainstream smoke and diluted sidestream smoke [1]. It is known to contain a variety of carcinogenic chemical compounds such as carbon monoxide and nicotine.

Passive smoking (PS) is ill-reputed to cause a number of health hazards in both adults and children. It is infamous for primarily causing lung cancer in adults. Other forms of cancers such as cancer of the larynx and nasopharynx and lymphoma are also a major cause of concern. In recent times, it has been linked to respiratory disease symptoms. Children are more vulnerable to the effects of passive smoking due to the presence of more red blood cells and faster breathing rates. They are usually exposed to PS by adults (parents or others), which can cause lung infections (bronchitis and pneumonia), frequent ear infections, increased frequency of sickness, cough, wheezing, shortness of breath, and other symptoms. Other diseases or discomforts caused by passive smoking include odor annoyance, nasal irritation, and allergies [2].

Salivary biomarkers, which can be measured in both healthy and diseased people, are sentinel molecules that could be used to monitor health and disease surveillance [3]. Tobacco products contain a high concentration of toxic chemicals, carcinogens, and free radicals in both stable and unstable forms. This can cause local irritation and tissue damage, which can result in the production of biomarkers [4]. The concentrations and levels of these biomarkers can be determined using specialized, designated reagents run in autoanalyzer or semi-autoanalyzer machines to interpret their concentrations and levels in a specific sample of that population. Such processes can aid in the diagnosis of various diseases and disorders, as well as their consequences for human health.

Alkaline phosphatase (ALP) is an intracellular hydrolase enzyme that is involved in cell metabolism. This isoenzyme group is found on the cell membrane’s outer layer. They assist in catalyzing the hydrolysis of organic phosphate esters found in extracellular space [5]. It has a crucial role in intracellular destructive processes and cellular damage. It has bicarbonate and phosphate ions, which help in buffering against acids [6]. It is usually measured in blood, urine, and saliva samples under the unit “IU/L,” i.e., “international units per liter.” A change in the levels of salivary alkaline phosphatase (S-ALP) can indicate a number of health issues. A rise in S-ALP levels denotes inflammation and healthy tissue destruction [7].

Although urine or serum samples are the most commonly used methods for ALP diagnosis, the use of saliva has some compelling arguments. When compared to serum or urine sample collection, salivary diagnosis is less invasive and faster. It is inexpensive and can produce results in as little as an hour. It includes no patient discomfort and easier sample handling for the healthcare professional [6].

S-ALP estimation can be used to identify tissue injury as an early biomarker. Identification of its levels in different gender and age groups will provide information about preventive measures that can be taken in passive smokers to avoid the effect of tobacco on tissue damage. The purpose of the present study was to compare S-ALP levels in passive smokers of various age groups, levels of exposure, and gender and compare it with that of healthy non-smokers.

## Materials and methods

Study design and setup

The present study was conducted in the outpatient department (OPD) of a private dental college and hospital situated in Navi Mumbai. Ethical clearance was obtained prior to the commencement of the study.

Sampling technique

The sample size was calculated using the formula for the finite population. After assuming a confidence level of 95%, margin of error of 5%, and a population proportion of 50%, we obtained 53 as the baseline to generalize the sample size. Therefore, a lower limit of 53 samples had to be selected for the study, and after rounding off as well as considering equal distribution between all the groups, 64 samples were included in the study.

Inclusion criteria

Patients with routine dental problems who came for treatment in dental OPD giving consent for participation in the study were included. Patients with passive smoking exposure for at least a year for the test group and participants who were not exposed to passive smoking ever were taken as the control group. Patients of all gender were included in the study.

Exclusion criteria

Patients with oral potentially malignant disorders and systemic illnesses such as diabetes, hypertension, thyroid or any known endocrine disorders, osteoarthritis, osteosarcoma, and renal failure were excluded. Patients who were diagnosed with periodontitis clinically based on probing depth, bleeding gums, and radiographic bone loss were also excluded from the study. Patients consuming alcohol or with any liver diseases were deferred. Patients with Paget’s disease, hyperparathyroidism, osteomalacia, metastatic bone disease, and a recent fracture were barred. Patients with any of these conditions were exempted from the study because these conditions are associated with altered alkaline phosphatase.

Choice of subjects and control

There were 64 samples included. Patients who visited the dental OPD for routine dental checkups and treatment for various dental ailments were considered, and their consent was obtained for inclusion in the study. A brief history and demographic data were gathered. To assess the exposure to passive smoking, a pre-study questionnaire/patient assessment sheet (PAS) was used (Appendices).

Grouping of age

Group 1 consisted of participants below the age of 14 years (childhood). Group 2 has participants aged between 15 and 25 years (young age). Group 3 had participants aged between 26 and 60 years (adulthood). Group 4 consisted of participants above the age of 60 years (old age). After inclusion in the study, the subjects were separated into test groups (passive smokers) and control groups (non-passive smokers).

Procedure

Subjects were informed about the purpose of the study followed by obtaining informed consent. They were instructed to fast for two hours prior to sample collection as it reduces alterations in salivary enzyme levels. The subjects were asked to collect the saliva on the floor of the oral cavity and spit it in the sample collection tube. A minimum of 2-2.5 mL of unstimulated saliva was collected from each subject. The samples were then centrifuged at 3,000 rotations per minute (rpm) for 10 minutes. The resultant 20 µL of supernatant was then mixed with 1,000 µL of ERBA Mannheim ALP reagent (Mannheim, Germany) and run in a semi-autoanalyzer. This was based on the kinetic method recommended by the International Federation of Clinical Chemistry. The reading obtained on the screen (in IU/L) was then noted and subjected to statistical tests.

Statistical tests used

Data obtained were compiled on a Microsoft Office Excel sheet version 2019 (Microsoft Corp., Redmond, WA, USA). Data were subjected to statistical analysis using the Statistical Package for the Social Sciences version 26.0 (IBM SPSS Statistics, Armonk, NY, USA). Frequencies and percentages were calculated for age and gender distribution. An intergroup comparison (between test and control) (two groups) was done using Student’s t-test. An inter-subgroup comparison was done using one-way analysis of variance (ANOVA). For all the statistical tests, p<0.05 was considered to be statistically significant, keeping α error at 5% and β error at 20%, thus giving a study power of 80%.

## Results

Demographic data

A total of 64 saliva samples were collected. Out of this, 32 were test (passive smokers) and 32 were control (healthy) samples. A total of 26 (13 test and 13 control) female participants’ saliva samples were included, and 38 (19 test and 19 control) male participants’ saliva samples were collected.

Comparison of S-ALP values in the test group and control group

An intergroup comparison of S-ALP values between the two groups (test and control) was done. There was a statistically highly significant difference seen between the S-ALP values between the test and control group (p<0.01) with higher values in the test group (Table 1). An inter-subgroup comparison was done to compare S-ALP values with age groups, gender, and time of exposure.

**Table 1:** Intergroup comparison of S-ALP values **Statistically significant (p<0.01) S-ALP: salivary alkaline phosphatase

S-ALP value	Number	Mean (IU/L)	Standard deviation (IU/L)	Standard error mean	t value	p value of t-test
Test	32	34.7213	11.80244	2.08640	9.931	0.000**
Control	32	12.0456	5.24742	0.92762		

Comparison of S-ALP values with age in the test group

There was a statistically non-significant difference seen for the S-ALP values in comparison with age groups in the test group (p>0.05) (Table 2).

**Table 2:** Comparison of S-ALP values with age groups in the test group #Statistically non-significant (p>0.05) S-ALP: salivary alkaline phosphatase, ANOVA: analysis of variance

Age groups	Number	Mean (IU/L)	Standard deviation (IU/L)	Standard error	95% confidence interval for mean		Minimum	Maximum	F value	p value of one-way ANOVA
					Lower bound	Upper bound				
Below 14 years of age	8	34.2125	11.64189	4.11603	24.4796	43.9454	25.61	58.65	0.053	0.984#
15-25 years of age	8	34.4538	14.34105	5.07033	22.4643	46.4432	22.00	55.10		
26-60 years of age	8	36.2100	11.08853	3.92039	26.9398	45.4802	22.60	54.00		
Above 60 years of age	8	34.0088	12.21654	4.31920	23.7955	44.2220	21.60	52.98		
Total	32	34.7213	11.80244	2.08640	30.4660	38.9765	21.60	58.65		

Comparison of S-ALP values with gender in the test group

While comparing S-ALP values of different gender in the test group, no statistically significant difference was found (p>0.05) (Table 3).

**Table 3:** Comparison of S-ALP values with gender in the test group #Statistically non-significant (p>0.05) S-ALP: salivary alkaline phosphatase

	Gender	Number	Mean (IU/L)	Standard deviation (IU/L)	Standard error	t value	p value of t-test
S-ALP value	Male	19	32.9053	11.47846	2.63334	-1.054	0.300#
	Female	13	37.3754	12.22156	3.38965		

Comparison of S-ALP values with time of exposure in the test group

On comparing the S-ALP values with time of exposure in the test group, we found a statistically highly significant difference (p<0.001) with higher values in individuals who were exposed to smoke for at least 30-60 minutes daily (Table 4).

**Table 4:** Comparison of S-ALP values with time of exposure in the test group **Statistically significant (p<0.01) S-ALP: salivary alkaline phosphatase

	Time of exposure	Number	Mean (IU/L)	Standard deviation (IU/L)	Standard error	t value	p value of t-test
S-ALP value	<30 minutes	27	31.8515	10.34790	1.99145	-3.840	0.001**
	30-60 minutes	05	50.2180	5.26937	2.35653		

## Discussion

Saliva has been extensively researched as a potential diagnostic tool over the last decade due to its ease of use and non-invasive accessibility, as well as its abundance of biomarkers such as genetic material and proteins [8]. It has the potential to enable chairside diagnosis of a variety of oral and systemic diseases [9]. It contains a diverse range of hormones, antibodies, growth factors, enzymes, microbes, and their products [10,11], allowing it to function as a promising and accurate diagnostic tool. Many of these constituents enter saliva via blood through passive diffusion, active transport, or extracellular ultrafiltration [10,12], making it a reflection of bodily physiological functioning [13]. It is a non-invasive technique that usually has better patient cooperation and is cost-effective and nearly as accurate as blood investigations.

As stated earlier, alkaline phosphatase is a group of intracellular hydrolytic enzymes participating in cellular metabolism [5]. It is mainly found in the liver and bones with trace amounts present in the kidneys, intestines, placenta, and leukocytes. The enzymatic group of ALP creates an alkaline environment that catalyzes the hydrolysis of phosphate esters. It also aids bone calcification metabolism as well as intestinal lipid transport, although its exact metabolic role is yet to be established [6,14]. The source of this enzyme in the oral cavity includes neutrophils, bacteria, and oral epithelial cells [15,16]. An increase in ALP levels is frequently linked to a number of diseases and conditions, including dental caries, periodontitis, malignant and potentially malignant disorders, smoking, and some bone diseases such as Paget’s disease. Patients with untreated celiac disease also show elevated levels. Low ALP levels are seen in patients with hypophosphatasia, postmenopausal women receiving estrogen therapy for osteoporosis, men who have recently had heart surgery, malnutrition, magnesium deficiency, hypothyroidism, severe anemia, children with achondroplasias, and cretinism [17].

The present study was conducted to find out S-ALP values in passive smokers and correlate them to different age groups, gender, and times of exposure. The mean values of S-ALP in the control group, i.e., healthy individuals, were found to be around 12 IU/L, which was similar to that obtained by Sushmitha et al. [18]. The mean S-ALP values obtained in the test group, i.e., passive smokers, were found to be 34.70 IU/L.

Studies done by Ibraheem et al. [19] and Hassan [20] showed that there was a rise in S-ALP levels in passive smokers in comparison to healthy individuals; however, the difference was statistically insignificant. In contrast to this, the present study’s results found that S-ALP levels were high in passive smokers when compared to healthy individuals and were statistically significant (p<0.01).

The present study’s objective was to evaluate the difference in S-ALP values in regard to age, gender, and time of exposure in individuals. We found no difference in S-ALP values in different age groups or gender. However, there was a statistically significant (p<0.01) difference seen in regard to time of exposure. S-ALP values were high in individuals who were exposed to smoke daily for around 30-60 minutes. Continuous exposure to smoke causes deleterious effects and leads to alteration in membrane-bound ALP levels. The effect of smoking or passive smoking on ALP may be due to an altered imbalance between the free oxygen radicals and the imbalance in the antioxidant levels [6].

Howard et al. [21], Howard et al. [22], and Chen et al. [23] have highlighted that passive smoking and active smoking have systemic effects in the same direction, although the magnitude may not be the same.

The use of saliva to detect the effect of passive smoking on health can help expand the literature on the utility of salivary alkaline phosphatase as a biomarker in assessing passive smoking effects. The current study’s results can indicate the effect of passive smoking in different age groups and gender, although the insignificant results may indicate the need for a larger sample size and more time to compare the results. Further studies with a larger sample size can help us understand the impact of passive smoking on tissue health more clearly.

Future recommendations

Future studies with larger sample sizes should be done to better understand the effects of passive smoking on tissue health. There is literature stating that passive smoking can lead to an increased risk of low-birth-weight pregnancy outcomes, and there is an increase in ALP levels in pregnant females exposed to smoke [24,25]. Studies can be done concentrating on S-ALP levels in such individuals, which would positively and effectively contribute to the literature as well as public health.

## Conclusions

The present study pointed out that there is a significant difference (p<0.01) between the S-ALP values of passive smokers and healthy individuals. This suggests that passive smoking has negative effects on body tissues. However, there exists no traceable difference (p>0.05) in the S-ALP values of different age groups and gender of passive smokers, but, to highlight, there exists a significant difference (p<0.01) in the S-ALP values in passive smokers based on their time of exposure to the smoke. This shows that continuous exposure to smoke may have harmful effects on healthy tissues, leading to an alteration in ALP levels. The study also showed that the S-ALP enzyme can act as a major non-invasive, accurate, and economical alternative in salivaomics.

## Figures and Tables

**Figure 1:**
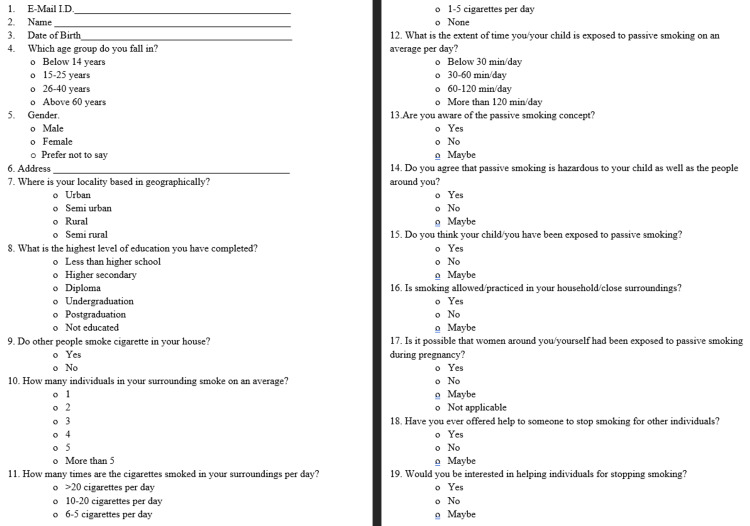
Patient assessment sheet

## Appendices

Figure 1 shows the pre-study questionnaire/patient assessment sheet (PAS) used in the present study.
